# Changes in Women’s Facial Skin Color over the Ovulatory Cycle are Not Detectable by the Human Visual System

**DOI:** 10.1371/journal.pone.0130093

**Published:** 2015-07-02

**Authors:** Robert P. Burriss, Jolyon Troscianko, P. George Lovell, Anthony J. C. Fulford, Martin Stevens, Rachael Quigley, Jenny Payne, Tamsin K. Saxton, Hannah M. Rowland

**Affiliations:** 1 Department of Psychology, Northumbria University, Newcastle, NE1 8ST, United Kingdom; 2 Centre for Ecology & Conservation, University of Exeter, Penryn Campus, Penryn, TR10 9FE, United Kingdom; 3 Division of Psychology, Abertay University, Dundee, DD1 1HG, United Kingdom; 4 School of Psychology & Neuroscience, University of St Andrews, St Andrews, KY16 9JP, United Kingdom; 5 MRC International Nutrition Group, London School of Hygiene and Tropical Medicine, London, WC1E 7HT, United Kingdom; 6 Department of Zoology, University of Cambridge, Cambridge, CB2 3EJ, United Kingdom; 7 Institute of Zoology, Zoological Society of London, London, NW1 4RY, United Kingdom; Brock University, CANADA

## Abstract

Human ovulation is not advertised, as it is in several primate species, by conspicuous sexual swellings. However, there is increasing evidence that the attractiveness of women’s body odor, voice, and facial appearance peak during the fertile phase of their ovulatory cycle. Cycle effects on facial attractiveness may be underpinned by changes in facial skin color, but it is not clear if skin color varies cyclically in humans or if any changes are detectable. To test these questions we photographed women daily for at least one cycle. Changes in facial skin redness and luminance were then quantified by mapping the digital images to human long, medium, and shortwave visual receptors. We find cyclic variation in skin redness, but not luminance. Redness decreases rapidly after menstrual onset, increases in the days before ovulation, and remains high through the luteal phase. However, we also show that this variation is unlikely to be detectable by the human visual system. We conclude that changes in skin color are not responsible for the effects of the ovulatory cycle on women’s attractiveness.

## Introduction

The females of several primate species advertise their ovulatory status through anogenital swelling [1–3], and facial or perineal skin color may also vary cyclically [4–7]. These changes attract male attention [4,8–10]. Although exaggerated sexual swellings were not the ancestral state in hominids [11], there is evidence that women’s social and sexual behavior does vary over the cycle [12–14] (cf. [15]). For example, near ovulation women are more attracted to masculine men [16,17], flirt more with attractive men [18], make greater efforts to augment their beauty, and choose to wear more revealing, fashionable, and red clothing [19–21]. There are also physiological cues to ovulation: the body odor [22–24] and voice [25–27] of women at periovulation are rated as more attractive. The possibility that facial appearance changes over the cycle has, until recently, received less attention [28], but men do rate facial photographs of unfamiliar women as more attractive when taken near ovulation [27,29,30] (cf. [31]). This may be due to changes in face shape [32–34], but a more likely mediator is hormone-related variation in skin color.

Estrogen and progesterone levels vary over the cycle [35]; the interaction between salivary estradiol and progesterone predicts women’s combined facial and vocal attractiveness to men [27].Women whose estrogen levels are especially high during their late follicular (fertile) phase are rated as more attractive, feminine, and healthy [36] (cf.[37]). The effects of cycle or hormones on appearance are likely explained by natural changes in the skin, because the effects emerge only when women are instructed to remove makeup before being photographed [27,29,30,36]. Women wear more makeup near ovulation [38] but researchers who permit makeup use find no effects of cycle or of late follicular hormone levels on facial attractiveness [32,36]).

Estrogen is implicated in many aspects of skin physiology, including aging, healing, hydration, hair growth, sebum production, and pigmentation [39–41]. The estrogen receptor ERβ is highly expressed in the epidermis, and particularly in the keratinocytes of the stratum basale [42,43], where melanocytes are concentrated [44]. Furthermore, in women whose estrogen levels are high, vascularization is greater and blood vessels more dilated [45,46]; this leads to more oxygenated blood [47] and redder skin [48]. Progestins are routinely prescribed as a treatment for acne vulgaris [49], and may therefore reduce redness. Although these studies do not document within-participant effects of cyclic variation in sex hormones, they do suggest direct pathways by which such variation could influence skin color and, consequently, attractiveness.

Although researchers have hypothesized a link between fecundability (the likelihood of conception during a specific time period) and skin pigmentation [50], the evidence for skin color change across the human ovulatory cycle is equivocal. Early studies indicated that women perceive darkening of their facial skin immediately prior to menstrual onset [51,52]. However, these results may be invalid because assessments were subjective and participants were aware of the studies’ aims. Snell and Turner [52] measured reflectance of forehead and cheek skin and quantified melanogenic activity in abdominal skin using biopsies; neither varied cyclically. These findings were replicated by Samson et al. [30], who took late follicular (high fertility) and mid luteal (low fertility) spectrophotometric measurements of cheek and forehead skin color and converted these to L*a*b* coordinates (a human visual color space). There was no effect of fertility status on any of the three color dimensions, leading the authors to conclude that “differences in men’s perceptions of attractiveness and healthiness [are] not driven by these measures”. Nevertheless, further investigation is warranted. Spectrophotometry may be unsuitable for the measurement of human facial skin color, as it requires the researcher to move into the participant’s personal space. This may elicit blushing [53], which would likely overshadow any less labile effects of cycle phase on skin redness. Also, spectrophotometry only permits analysis of small point samples (mm in size), and so is prone to missing overall changes in color.

Photography has several advantages over spectrophotometry. It is fast, allows for distance between the researcher and the participant, and permits analysis of a larger area of skin rather than a limited number of point samples. Oberzaucher et al. [33] took photographs of women and extracted mean red, green, and blue (RGB) color values from cheek patches. They found that skin is redder at periovulation than during the luteal phase. However, RGB values from photographs do not represent color as it appears in the real world and when processed by the human visual system. Under factory settings cameras respond nonlinearly to light intensity and are biased toward certain wavebands, particularly the long (red) [54]. Because Oberzaucher et al. [33] neither report correcting for these problems, nor state how the color changes they describe would be perceived by humans, the effect they identify may be inaccurate or, even if genuine, so small as to be biologically irrelevant [4]. Recently, Jones et al. [55] reported data from two samples showing significantly greater redness, but not yellowness or lightness, of facial skin when women are photographed at times when their salivary estradiol is relatively high. They used a 24-colour chart to convert their images from non-linear camera RGB to CIELAB values. It is difficult to assess the effectiveness of their conversion, particularly given the possibility of eye-camera metamerism, which is far more likely when such a small sample of colours is used for calibration. We note that 40 to 60 samples is recommended for this technique [56]. Jones et al. [55] argue that the changes they detect may be visible to humans, given that discrimination thresholds for within-participant changes in facial redness are lower than for non-face stimuli [57], but they do not estimate perceptibility with a model of the human visual system.

In this study we use a method outlined in detail by Stevens et al. [54],[58] to objectively measure the color of skin patches from photographs and map color values to the human visual system, thereby enabling accurate representation of color and quantitative measurement of perceptual differences [59]. These methods have been applied to the study of cyclic variation in facial skin color in the rhesus macaque, demonstrating that the ratio of red to green (hereafter, redness) is higher [5] and luminance lower [4] when females are most fertile. Because there is no single consistently applied method of categorizing human fertile and nonfertile phases [60], and because it is unclear at which points in the cycle one might expect the greatest variation in skin color [33,55], we opted to photograph women daily for the duration of at least one cycle and to analyze data using Fourier regression. This analytical method is more commonly employed in the fields of epidemiology and climatology [61,62], but is suitable here because our outcomes vary continuously over a cycle of known periodicity (the ~28 day ovulatory cycle). We hypothesized that skin redness and luminance peak near ovulation and are lower at other times, and that these differences would be perceptible to the human visual system.

## Methods

### Ethics statement

This study was approved by the Cambridge Psychology Research Ethics Committee (project number pre.2011.66). Participants consented in writing before taking part.

### Participants

We recruited 30 female participants through the social contacts of two female undergraduate researchers at a UK university. Three participants withdrew and five were excluded (three for not detecting a luteinizing hormone surge, and two for not reporting the date on which their next period began). The final sample was 22 women (mean age = 23.36 years, SD = 4.94). Eighteen self-identified as White, three as East-Asian, and one as Hispanic. We did not ask participants to identify their sexuality.

Participants consented to participate in a study of appearance and to be photographed daily, excluding weekends, for the duration of at least one month. We did not inform participants of our hypotheses. At the start of the study, no participant had used hormonal contraceptives for at least three months [30,36,63]. Hormonal contraceptive users were excluded because research indicates that the attractiveness of these women does not change cyclically [24,25,64], and because we expected that hormonal contraceptives would disrupt any cyclical effects of hormones on skin color. Other studies that report cyclic changes in appearance have excluded women who use hormonal contraceptives [27,29,30,55].

Participants reported average cycle duration, date of onset of current or previous menses, and the expected date of onset of their next menses. Three days before the estimated date of ovulation (following Puts [65]), participants began daily use of luteinizing hormone (LH) tests with urine applicators (Clearblue Easy Ovulation Test; Unipath, Bedford, UK). Participants received verbal and written directions so that they could interpret the test results. We did not inform participants what the tests were designed to detect, nor did we provide the tests in original packaging. Participants reported the date on which their test results were consistent with a surge in LH. After detecting a surge, participants continued to test for at least two days to verify the end of the surge (multiple peaks or a prolonged surge may indicate an abnormal cycle [66]). Participants reported the onset of next menses, either in person or via email. At debrief, no participant reported having correctly intuited the hypothesis of the study.

### Photography

Photography took place during UK winter months (January 2012 through March 2012, and November 2012 through March 2013) when tanning through exposure to the sun is likely to be minimal. All photographs were taken by women (RQ, JP, or HMR), because the sex of the researcher can influence participant facial temperature [53] (if increases in temperature are associated with blushing, the sex of the photographer may also affect participant skin color). We arranged for photographs to be taken on weekdays, as and when participants were available (mean number of photographs analyzed per participant = 13.36, SD = 4.11). Participants were photographed at the same time each day, either between 14:00 and 15:00 or 18:00 and 19:00, and reported removing makeup at least one hour prior to arriving at the laboratory. We asked participants to adopt a neutral expression and head posture, remove spectacles, and tie their hair back from their face and ears. Participants wore a black hairdressers’ smock to limit the effects of clothing on appearance, because light reflecting off clothes may cast a noticeable tint on skin tones (color spill). This is especially important given evidence that women’s choice of clothing type and color varies cyclically [19–21]. Although some researchers investigating facial skin color have taken similar precautions to mitigate possible effects of clothing on appearance [67], Jones et al. [55] is the only other study of cycle and appearance to report doing so.

Photography took place in one of two small rooms with drawn curtains. The participant sat before a beige felt backdrop onto which was affixed an 18% gray card. Our camera was a Canon 350D camera with Canon zoom lens EFD 18-55mm. A ring flash (Canon macro ring lite MR-14EX flash) was used to ensure even lighting on the participant’s face. The camera was placed 2m from the participant’s chair, and tripod height was adjusted so that the camera lens was level with the participant’s eyes. Photographs were taken in RAW format to avoid lossy compression [68]. The photographer inspected all photographs immediately and retook any that were unsatisfactory (due to technical problems or to the participant blinking, tilting her head, or adopting a non-neutral expression).

### Color measurements

Camera linearization models were generated from eight calibrated Spectralon grey reflectance standards varying in reflectance from 99% to 2% [54]. Linearization models for red, green, and blue had R^2^ values >0.999. Photographs were converted to uncompressed 16-bit TIFF files, and then linearized and standardized against the 18% grey card using a script written by JT in ImageJ [69]. This process controls for the effect of any variation in light conditions [54].

**Fig 1:**
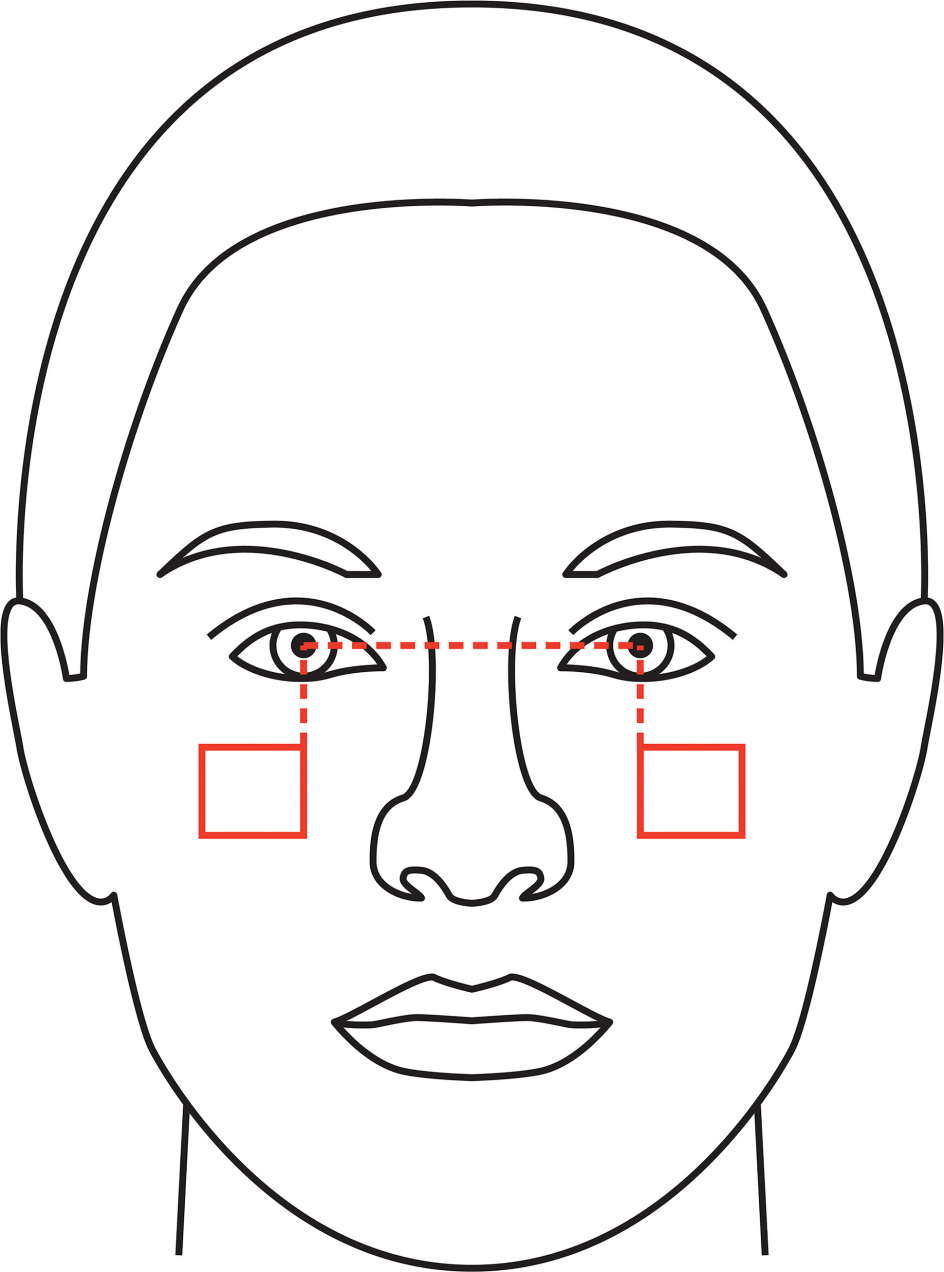
Location of skin patches extracted from each photograph for use in color analyses. Dashed lines are distances used to position patches; solid rectangles describe the patches. See text for procedure.

We extracted two skin patches from each photograph in ImageJ, one on each cheek. Patch width was 30% of the participant’s interpupilary distance, and patch height 26% of the interpupilary distance. The innermost top corner of each patch was positioned 30% of the interpupilary distance below the mean Y coordinates of the pupils and on a line bisecting each pupil on the X axis (see Fig 1). Patches were therefore equivalent in relative dimensions and position to those extracted by Jones et al. [70].

We measured the mean RGB values for each patch, and converted these to photon catch values equivalent to long, medium, and short wave (LMS) cone responses, and to CIE XYZ responses. We then averaged the left and right patch values, giving one color value per photograph. Cone-catch models were generated following the methodology of Párraga et al. [59]. Human cone-catch quanta (LMS sensitivities from Stockman and Sharpe [71], and CIE XYZ 1931 standard 2° observer) and camera responses were calculated from a dataset of 3139 natural reflectance spectra, modeled under D65 lighting conditions at 1nm increments from 400-700nm. Polynomial models were generated from these cone-catch quanta allowing us to map from camera to human cone-catch values. All models reported conversion R^2^ values > = 0.999. Mapping from camera to animal color space is highly accurate compared to modeling animal vision with reflectance spectra (e.g. [72,73]).16-bit RGB images were converted to CIE XYZ and then to LAB in 32-bit to rule out clipping. All model generation and image processing was performed using custom-written code (JT) in R [74] and ImageJ [69]. Human LMS color differences were calculated as just noticeable differences (JNDs) using a model of receptor noise by Vorobyev and Osorio [75], where a value of 1 corresponds to a discriminable or perceptible difference under optimal lighting conditions. We calculated JNDs using cone ratios of 1:0.5:0.03125 SW:MW:LW, and Weber fractions of 0.08:0.02:0.02 and 0.09:0.02:0.02) based on Vorobyev and Osorio [75]. Because cone ratios are variable in humans we also calculated JNDs using the minimum and maximum cone ratios reported for humans by Hofer et al. [76] of 1:1:0.03125 and 1:0.06:0.03125 SW:MW:LW. In addition to JNDs we calculated perceptual differences in LAB space, an internationally developed color space that is the standard for representing human color vision. In LAB space, L specifies luminance (achromatic brightness), A specifies the red-green ratio, and B specifies the blue-yellow ratio. A difference greater than 2.2 of any values within one of these three axes is noticeable under optimal lighting [77]. Cheek patch color contrasts were calculated against day 14 of the adjusted cycle (the day of the luteinizing hormone surge; see below), generating JND differences, and differences in the A (redness) axis values throughout the cycle (A_diff_; i.e. day 14 A value minus the sample day value). We also calculated differences in the B axis and Euclidean distance between the three points (∆E) ((S1 Text and S1 and S2 Figs).

### Fertility estimation

Estimates of mean cycle duration and the timing of ovulation and the LH surge vary [35,66,78–82]. We assume a mean cycle duration of 28 days [78,81] and that, in a 28 day cycle, the urinary LH peak occurs one day before ovulation [81] and 15 days prior to menstrual onset [80] (i.e. on day 14 of a 28 day cycle, where menses begins on day 1).

Participants’ cycles differed in length. Following Puts [65], we fit all participants to an ‘adjusted’ 28 day cycle. First, we numbered each cycle day *D* ^*n*^, beginning with the first day of menses (*D* ^*1*^). Then we transformed *D* ^*n*^ into their expected equivalents in a 28 day cycle (*D*_*a*_^*n*^): the day of onset of menses was coded *D*_*a*_^*1*^, the day of the LH surge as *D*_*a*_^*14*^, and the final day of the cycle (the day preceding the onset of the next menses) as *D*_*a*_^*28*^. Other days were transformed such that, for days preceding the LH surge, *D*_*a*_^*n*^ = (13 / (*D* ^*lh*^—1)) + *D*_*a*_^*p*^, and for days succeeding LH surge, *D*_*a*_^*n*^ = (14 / (*D* ^*f*^—*D* ^*lh*^)) + *D*_*a*_^*p*^, where *D* ^*lh*^ is the *D* ^*n*^ of the LH surge, *D* ^*f*^ is the *D* ^*n*^ of the final day of the cycle, and *D*_*a*_^*p*^ is the *D*_*a*_^*n*^ preceding the *D*_*a*_^*n*^ being calculated. For example, if cycle duration is 33 days and the LH surge occurs on *D* ^*20*^, the *D*_*a*_^*n*^ of the second cycle day (*D* ^*2*^) would be (13 / (20–1)) + 1 = 1.68, and the *D*_*a*_^*n*^ of the day succeeding the day of the LH surge (*D* ^*21*^) would be (14 / (33–20)) + 14 = 15.08.

Following Gangestad et al. [83–85], we estimated women’s conception risk at each session using actuarial data on the likelihood of conception after a single act of intercourse in women with regular cycles [86]. We assigned risk values based on the participants’ transformed cycle days, interpolating between conception risk estimates where transformed days were not integers (e.g. a photograph taken on *D*_*a*_^*10*.*5*^ would be allocated a value midway between those associated with days 10 and 11).

### Statistical analyses

**Fig 2:**
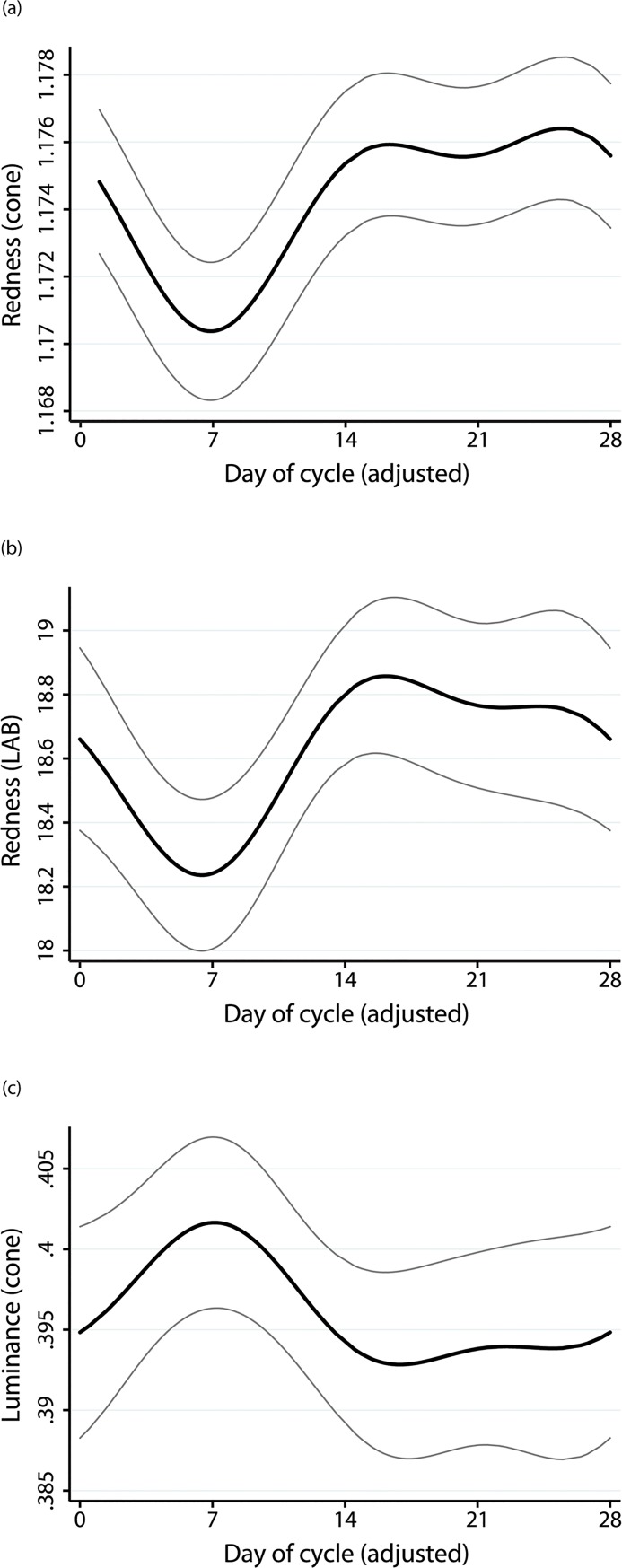
Color of facial skin over the 28-day adjusted ovulatory cycle. Mean redness (a) and luminance (c). (b) shows redness in LAB space. The grey lines indicate 95% confidence intervals. Higher values mean redder or lighter skin. The variation in redness is significant; the variation in luminance is not.

We carried out the statistical analysis in Stata 12 [87], employing a mixed effects Fourier (or trigonometric) regression model. Fourier regression is a natural extension of cosinor-rhythmometry developed by Nelson et al. [88] to analyze how an outcome varies continuously over a cycle of known periodicity. Whereas cosinor-rhythmometry fits only a simple sine wave, Fourier regression has greater flexibility to capture more realistic cyclic patterns. As far as we are aware, Fourier regression has not previously been used in research on the human ovulatory cycle, but it is widely used to analyze seasonality and diurnal rhythms in epidemiology and climatology (Fernández et al. [61] and Bliss [62] provide instructive examples) and is regarded as the standard approach for this type of data [89]*—*the circular equivalent of polynomial regression. Random intercepts were fitted to account for variation between participants and, nested within participant, between cycles. The cyclic patterns were captured using the first two pairs of Fourier terms, i.e. sin(θ), cos(θ), sin(2θ) cos(2θ), where θ is the angle representing the cycle phase at the time of measurement. (Higher order terms were not found to be significant.) The effect size was measured as the standard deviation of the fitted curve over the full cycle [90], i.e. the square root of half the sum of the squared coefficients of the Fourier terms. We based the confidence intervals for the fitted curves plotted in Fig 2 on the variances and covariances of the parameter estimates obtained from the observed information matrix. This tells us about the precision with which the shape of the curves were estimated while ignoring the (irrelevant here) precision with which the intercept (mean across all women) is measured.

## Results

The ratio of long wave to medium wave cone responses (i.e. redness) varied significantly across the ovulatory cycle, *χ* ^*2*^ (4) = 18.02, *p* > *χ* ^*2*^ = 0.0012. Redness decreased rapidly after menstrual onset, increased during the second week of the cycle, and remained elevated throughout the luteal phase (Fig 2A). Controlling for conception risk did not alter the significance of the cycle effect, *χ* ^*2*^ (4) = 14.79, *p* > *χ* ^*2*^ = 0.0052.

This variation in redness (chromatic difference) was not, however, predicted to be perceptible to the human visual system when we modeled JNDs using the Vorobyev and Osorio model with three different cone ratio values representing standard, minimum, and maximum cone proportions. All JNDs were <1. Nor was the variation in redness perceptible as measured by the A axis of LAB space. The change in redness did not exceed 2.2 in magnitude (Fig 2B, amplitude change is about 0.6 +/- 1.2). A difference of 2.2 in LAB space equates to a perceptual difference [77].

Luminance varied across the ovulatory cycle, but not significantly (Fig 2C).

## Discussion

We predicted that the redness and luminance of women’s facial skin would peak near ovulation. Contrary to our hypothesis, cyclic changes in luminance were non-significant. Redness did vary significantly across the ovulatory cycle, but the pattern of change was more complex than we anticipated. The ratio of long wave to medium wave (LW:MW, red:green, high values signify redness) decreased during the first week of the cycle, before increasing in the days preceding ovulation when conception after a single act of intercourse is most likely [86,91]. Redness remained elevated throughout the nonfertile luteal phase. However, these changes were small; calculation of discrimination thresholds using two models of human vision (LAB space and receptor noise) indicated that individual differences would not be detectable even under optimal lighting conditions. It is therefore doubtful that cyclical changes in skin color drive the reported effects of cycle on women’s appearance.

When women are in the fertile rather than the nonfertile phase of their cycle, their faces are rated more attractive [27,29,30,33]. Roberts et al. [29] found that masking hair, ears, and visible clothing reduces but does not eliminate this effect, demonstrating that some of the variance in attractiveness is due to changes in the face itself. These changes could involve face shape and expression [32–34] or, as Roberts et al. [29] suggest, lip color and size, pupilary dilation, and skin color and tone [29]. Humans are sensitive to variation in skin color [57,92], and are attracted to facial skin color patterns characteristic of healthiness [70], youthfulness [93], a diet rich in carotenoids [94,95], and high blood perfusion [96].

Our findings are in line with those of Oberzaucher et al. [33] and Jones et al. [55], in that we found that human facial skin varies cyclically in redness but not luminance. Oberzaucher et al. [33] analyzed photographs taken on the day of ovulation (high fertility) and 14 days after ovulation (low fertility). Our results, from photographs taken daily over the whole cycle, suggest that redness remains high and constant between ovulation and the onset of the next menses, and so do not support the pattern of change reported by Oberzaucher et al. [33]. We believe that our findings are the more valid because we used methods that give an accurate measure of real-world color as perceived by the human visual system. Jones et al. [55] photographed women five times at intervals of one week and found that facial skin was redder when estradiol levels were relatively high. They note that estradiol is high during the late follicular phase but can remain high during the early luteal phase [97]. These are the phases when we found skin to be at its reddest.

When we modeled how skin would be perceived by the human visual system, we found that, although significant changes in redness were detected, the differences in skin color were below the level detectable by the human visual system. Even if redness comparisons were made by persons with maximally sensitive cones of individuals displayed side-by-side under ideal lighting conditions, the differences would not be noticeable [77]. It is therefore unlikely that these genuine color differences act as a cue or signal of female fertility status, or are responsible for effects of cycle on female attractiveness [27,29,30,33,98]. Nevertheless, our results must be considered preliminary until we have established through behavioral testing of human receivers that the change in redness is not detectable.

We also did not find an effect of cycle on facial skin luminance, suggesting that self-reported changes in human skin lightness are inaccurate or based on regions of the face (e.g. around the eyes) that we did not analyze here [51,52]. The relationship between cycle and skin luminance seen in the rhesus macaque [4] does not appear to be a feature of our own lineage.

Women may lack a perceptible skin color cue to their fertility status because they have lost what was once an advertisement of ovulation, possibly to confuse males as to the paternity of their offspring [99] or for other reasons unrelated to sexual selection [100]. Cyclical changes in skin color in the absence of anogenital swellings may by symptomatic of an evolutionary stage preceding loss of all fertility-related skin color changes [5]; if we ever possessed these color cues, our species may be nearer to complete loss than other catarrhines. Evidence shows that anogenital swelling evolved in our closest primate relatives only after our lineages diverged [101,102], and therefore that humans never possessed this more conspicuous cue to fertility. Women may stand to benefit by concealing all remaining cues to ovulation, such as body odor [26,27] or face shape [32–34], but have not yet concealed these sufficiently to avoid their being detected by men, who are (or were) under selection pressure to acquire information about female fertility [12,28]. Alternatively, women may have suppressed cues to ovulation that are widely perceptible, and retained those that can be directed at preferred men. Women’s voices are more attractive at peak fertility [25,26], but women also modulate their voices to sound more attractive when addressing attractive men [103]; it remains to be seen whether this modulation is greater midcycle. Attractive physical ornaments, such as revealing or red clothing [19–21], may also be adopted when women are likely to encounter attractive prospective mates. And there is evidence that attractive behaviors, such as a flirtatious manner, are deployed more at peak fertility—but only in the presence of attractive men [18]. This lends support to the suggestion by Campbell [104] that researchers might find “voluntary signaling by the female replacing the involuntary physiological signals of estrus”. Some physical cues may, however, be equally labile as the behavioral and be facultatively deployed. Men are attracted to women with dilated pupils [105], which indicate arousal. Women might attract unwanted male attention if their pupils were permanently dilated, or if they were dilated for the duration of the fertile phase. As pupil size can vary on the scale of seconds, it is unsurprising that women’s pupils increase in diameter during the fertile phase, but only in response to sexually significant stimuli [106]. Skin redness can also vary rapidly, as when a person blushes and the skin is perfused with blood [107]. Although we found an effect of cycle on skin redness, it is possible that skin redness may, like pupil dilation, be perceptibly greater during the fertile phase only in response to sexually significant stimuli.

A limitation of our study is that our sample mostly comprised White women. All of the research on cyclic variation in facial appearance has involved predominantly White/Caucasian samples from Europe or North America [27,29–34]. Research that replicates the effect of cycle on attractiveness in non-White samples may be informative. Estradiol levels are higher at all points of the cycle in African American compared to White American women [108]. As estrogen is implicated in cyclic variation in phenotype [27,55,63,109,110], facial attractiveness may vary differently in women of different ethnicities. This may be especially true of skin color variation, because Black African observers rely more on skin color when judging the attractiveness of Black African faces, while White Europeans rely more on face shape [111].

Further investigation is also warranted to determine the relationship between cyclic variation in skin redness and basal body temperature (BBT). The pattern of change we report is not what one would expect if redness is influenced primarily and directly by estrogen, which peaks in the days preceding ovulation but is relatively low during most of the luteal phase [112] (cf. [97]). BBT abruptly rises after ovulation and remains high until the onset of the next menses [113]; this also describes the change we observed in skin redness, except that skin redness tended to rise prior to, rather than subsequent to, ovulation. It is plausible that skin becomes redder around ovulation because blood flow to the skin increases to allow heat to be convected from the body. Future studies should investigate if cyclic variation in facial skin redness is greater in women who vary more in BBT, and whether the change in skin redness precedes that in BBT.

This study shows that human female skin redness varies over the cycle but that this variation is not perceptible by the human visual system. We therefore conclude that the well-documented cyclic variation in female facial attractiveness is not driven by color. Whether men’s responses to women who differ in cycle position and are encountered outside of the laboratory [64,98] are influenced by women’s facultative variation in skin color, possibly mediated by body temperature [53] and blood perfusion/oxygenation [96], is an outstanding question.

## Supporting Information

S1 Dataset Includes cycle day (adjusted), and cone LW, MW, and SW. (XLSX)

S1 Fig ∆E of facial skin over the 28-day adjusted ovulatory cycle. The shaded area shows +/- 1 standard error. ∆E changes encompass both color and luminance differences, and cheek patch values were found to vary significantly throughout the cycle. However, the magnitude of the modeled change (0.43) would not be perceptible to humans even if the samples were presented side-by-side under optimal conditions. S2 Fig reveals that the majority of the differences expressed in ∆E were due to red-green ratio changes rather than L (luminance) or B (blue-yellow).
(EPS)

S2 Fig A_diff_ of facial skin over the 28-day adjusted ovulatory cycle. The shaded area shows +/- 1 standard error. Cheek patch redness (red-green ratio) varied linearly with cycle day, from more red-shifted on day 0 to more neutral-shifted on day 28. However, this change (0.89) would not be perceptible by humans under ideal lighting conditions.
(EPS)

S1 Text Additional analyses. (DOCX)
