# Supplementary material for: Changes in Women’s Facial Skin Color over the Ovulatory Cycle are Not Detectable by the Human Visual System
Source: PLoS One. 2015 Jul 2;10(7):e0130093. doi: 10.1371/journal.pone.0130093 (PMC4489916; doi:10.1371/journal.pone.0130093)
Supplement: S1 Text — (DOCX) [file pone.0130093.s004.docx]

**S1 Text, Additional analyses**

## Statistical Methods

Statistical tests were performed in R v3.0.2 [1]. Due to the repeated measures design, linear mixed effect models were specified using the LME4 package v1.1-5 [2] with a Gaussian error structure and fitted with restricted maximum likelihood. L_diff_, A_diff_, and B_diff_ and Euclidean distances (∆E) for each participant relative to *D_s_^14^* of their cycle were specified as response variables, with cycle-adjusted day and conception risk as fixed effects, each fitted with 3^rd^ order polynomials in full models, and participant specified as a random effect. Full models were then simplified with the fitLMER function of the LMERConvenienceFunctions v2.5 using AIC and log-likelihood to backwards-fit the fixed effects [2]. Model residuals were checked to verify assumptions of homogeneity of variance and a normal error structure, and variables were transformed to meet these assumptions where necessary [3]. Conservative degrees of freedom were used to calculate *p*-values from maximum likelihood models using the R function pamer.fnc. Final models were also compared to null models (with no fixed effects) using ANOVAs, with full maximum likelihood models.

## Results

∆E varied with a 2^nd^ degree polynomial of day, conception risk was removed from the simplified model (final model log∆E ~ poly(day, 2) + (1|participant), F_2,231_= 4.47; *p* = 0.012, deviance explained = 2.85%, the simplified maximum likelihood model was a better fit than the null, *p* = 0.012, effect size = 0.43 with the lowest difference on day 17, and highest on day 0, see Figure S1). L_diff_ was not found to vary with day or conception risk, all terms were removed from the simplified model, and were not a better fit than the null (*p* > 0.05). A_diff_ varied linearly with day, conception risk was removed from the simplified model (final model: A_diff_ = day + (1|participant), F_1,232_= 12.14; *p* < 0.001; deviance explained = 3.06%, the simplified maximum likelihood model was a better fit than the null, *p* < 0.001, effect size = 0.89 with the difference decreasing with cycle day, see Figure S2). B_diff_ was not found to vary with day or conception risk, all terms were removed from the simplified model, and were not a better fit than the null (*p* > 0.05).

**References**

1. R Core Team. R: A Language and Environment for Statistical Computing. Vienna, Austria: R Foundation for Statistical Computing; 2013.

2. Baayen RH, Davidson DJ, Bates DM. Mixed-effects modeling with crossed random effects for subjects and items. Journal of Memory and Language. 2008;59(4):390-412. doi: 10.1016/j.jml.2007.12.005.

3. Zuur A, Ieno EN, Walker N, Saveliev AA, Smith GM. Mixed effects models and extensions in ecology with R: Springer; 2009.
